# A comparison of intraocular pressure measurement using SUOER SW-500 rebound tonometer and conventional reusable Goldmann prisms

**DOI:** 10.3389/fmed.2024.1269332

**Published:** 2024-06-13

**Authors:** Jia Quan Chaung, Thanendthire Sangapillai, Karen Kate Quilat, Shamira Perera

**Affiliations:** ^1^Singapore National Eye Centre, Singapore, Singapore; ^2^Singapore Eye Research Institute, Singapore, Singapore; ^3^Duke-NUS Medical School, Singapore, Singapore

**Keywords:** glaucoma, rebound tonometer, IOP, intraocular pressure, Goldmann applanation tonometry

## Abstract

**Introduction:**

To determine the agreement between intraocular pressure (IOP) measurements using conventional Goldmann applanation tonometry (GA^1,2^T) and SUOER SW-500 Rebound Tonometer.

**Methods:**

This was a retrospective observational study where 205 eyes of 106 glaucoma patients had their IOPs measured by 2 fellowship trained ophthalmologists. Data were analyzed using the Bland–Altman method of differences. Correlation was measured using the Pearson coefficient.

**Results:**

Most of our patients were Chinese (88.7%) and female (51.9%). The average age was 66.9 years. The range of IOPs as measured by GAT was 2 to 58 mm Hg. Using the Bland–Altman method to compare GAT and SUOER SW-500 Rebound Tonometer. The tonometer overestimated the IOP by 0.5 mm Hg in the right eye and underestimated it by 0.1 mm Hg in the left eye. Overall, the tonometer overestimated the IOP by 0.2 mmHg. The Tonometer IOP correlated well with GAT, with a Pearson coefficient of correlation(*r*) of 0.89 (*p* < 0.001) for the right eye and 0.86 (*p* < 0.001) for the left eye, respectively. In patients with GAT IOP ≥ 21 mm Hg (*n* = 25), the Tonometer underestimated the IOP by 2.96 mm Hg.

**Discussion:**

The IOP measurements from the SUOER SW-500 Rebound Tonometer correlates well with the conventional GAT in measuring the IOP within normal ranges of IOP. SUOER SW-500 Rebound Tonometer may be of use, especially if the risk of transmission of infection is high considering that the probes are disposable. It is easy to use and its small size and portability makes it useful in situations where the patient is unable to be examined at the slit lamp.

## Introduction

Goldmann applanation tonometry (GAT) has been the gold standard for measuring intraocular pressure (IOP) for decades. IOP is the major modifiable risk factor for glaucoma and its measurement is integral for appropriate management. Treatment of glaucoma is mainly directed at lowering IOP. However, not all patients are able to be examined under a slit lamp and hence may not be suitable for GAT.

The SUOER Rebound Tonometer (RBT; model SW-500) is a hand-held, lightweight and contact tonometer that measures IOP with the help of a disposable probe. The motion parameters of the probe is recorded through an induction based coil system. The deceleration of the probe is analysed. The deceleration speed correlates with IOP. For example, the higher the IOP, the faster the deceleration of the probe and the shorter is the duration of impact.

A number of studies have compared the accuracy of different portable RBT, in particular between the GAT and versions of the Icare (e.g. IC100, IC200, Care Pro, TA01i) which are similar in design to the SUOER SW-500 Tonometer. However, these studies have published rather inconsistent results. Some studies reported under measurement of IOP by the Icare in comparison to GAT (1, 2). Other studies found Icare overestimates IOPs when compared to GAT (3–5). While other studies have also reported no significant differences in mean IOP measured by Icare compared to GAT when the IOP measured was in the normal ranges (6, 7) and even at extremes of IOP (8). The cause of these differences are uncertain, it is likely that there may be a multiple factors contributing to this difference. This includes the variability in the patients’ age range, sample size of study, study bias, ethnicity, previous glaucoma filtration surgery, whether they were healthy subjects or subjects with pre-existing glaucoma. Moreover, external factors such as central corneal thickness, corneal astigmatism and even altitude can affect the reliability of IOP measurement (9, 10).

The advantage of RBT over GAT is its portability and that it does not require topical anesthetics or fluorescein staining. Its portability allows IOP measurements in situations that the GAT may be difficult to use such as in young children, in the operating room, bed-bound patients. The disposable probes also reduces cross contamination. As IOP measurements with the RBT does not require topical anesthetics, it also reduces the risks of damaging the corneal surface while measuring IOP.

To this date, it is our understanding that there are no published studies that compares the performance of the SUOER SW-500 Rebound tonometer against the gold standard GAT. As the SUOER SW-500 rebound tonometer may be a potential portable screening tool for IOP, we have decided to compare its accuracy in IOP measurements against the GAT.

## Materials and methods

### Patient selection

One hundred and six patients that underwent follow-up visits at the Singapore National Eye Centre Glaucoma Clinic were included in our study. The principles of the declaration of Helsinki were adhered to and approval from the Singapore Eye Research Institute’s Institutional Review Board was obtained for a retrospective review of these prospectively collected cases. Patients were excluded if they were under 21 years of age, had corneal abnormalities that might render IOP measurements inaccurate (severe epithelial/stromal edema, large central scars), patients with corneal dystrophies, previous corneal refractive surgery or corneal transplantation, active ocular infection, poor cooperation, or refused to participate. Prior corneal refractive surgery as well as corneal transplants affects the overall reliability of IOP measurements (11–13). Furthermore, since corneal thickness was not measured, these patients were excluded.

### IOP measurement

After instillation of proparacaine hydrochloride 0.5% and application of sterile fluorescein 10% strips, IOP was measured on a slit lamp biomicroscope for both eyes of each participant. The IOP of the right eye was measured first using conventional GAT followed by SUOER SW-500 rebound tonometry. Subsequently, the IOP of the left eye was measured using SUOER SW-500 rebound tonometry followed by GAT. These measurements were taken at the same clinic setting and at the same time. IOP measurement was performed by 2 fellowship trained ophthalmologists. As previous research has reported that repeat tonometry induces a decrease in IOP and that there is a consensual decrease in IOP in the other eye (14) our methodology eliminated this bias by not using GAT readings more than once per eye.

The study used a separate IOP measurer and reader and by having the tonometer reset to 10 mm Hg before each reading. One person adjusted the dial in a masked manner, and a second person recorded the value.

### Statistical analysis

In terms of sample size calculation, a sample of 64 patients was needed (*α* = 0.05, *β* = 0.80 and S.D. 2.0 mmHg). Sample size was estimated with the G*Power program (version 3.1.9.6, University Dusseldorf, Germany). All statistical analyses were performed using Statistical Package for the Social Sciences version 29.0 (SPSS Inc., Chicago, IL). Normality check of data was performed with Kolmogorov–Smirnov and Shapiro–Wilk Tests. Both IOP measured by GAT and SW-500 Rebound tonometer were not normally distributed (*p* < 0.001). We plotted the differences between the 2 methods against their average (the Bland–Altman method of differences). The Pearson coefficient of correlation was calculated for each eye.

### Results

There were 106 (205 eyes) participants of which 51 were male and 45 were female (Table 1). The race proportion was 88.7% Chinese, 3.8% Malay, 5.7% Indian, 1.9% Other Ethnicities. The average age was 66.9 years (range, 26 to 89 years).

**Table 1:** Demographics of glaucoma clinic patients.

Female Sex (%)	51.9
Mean age (*y*) (range)	66.9 (26–89)
Race (% Chinese, % Malay, % Indians, % others)	88.7, 3.8, 5.7, 1.9

**Figure 1:**
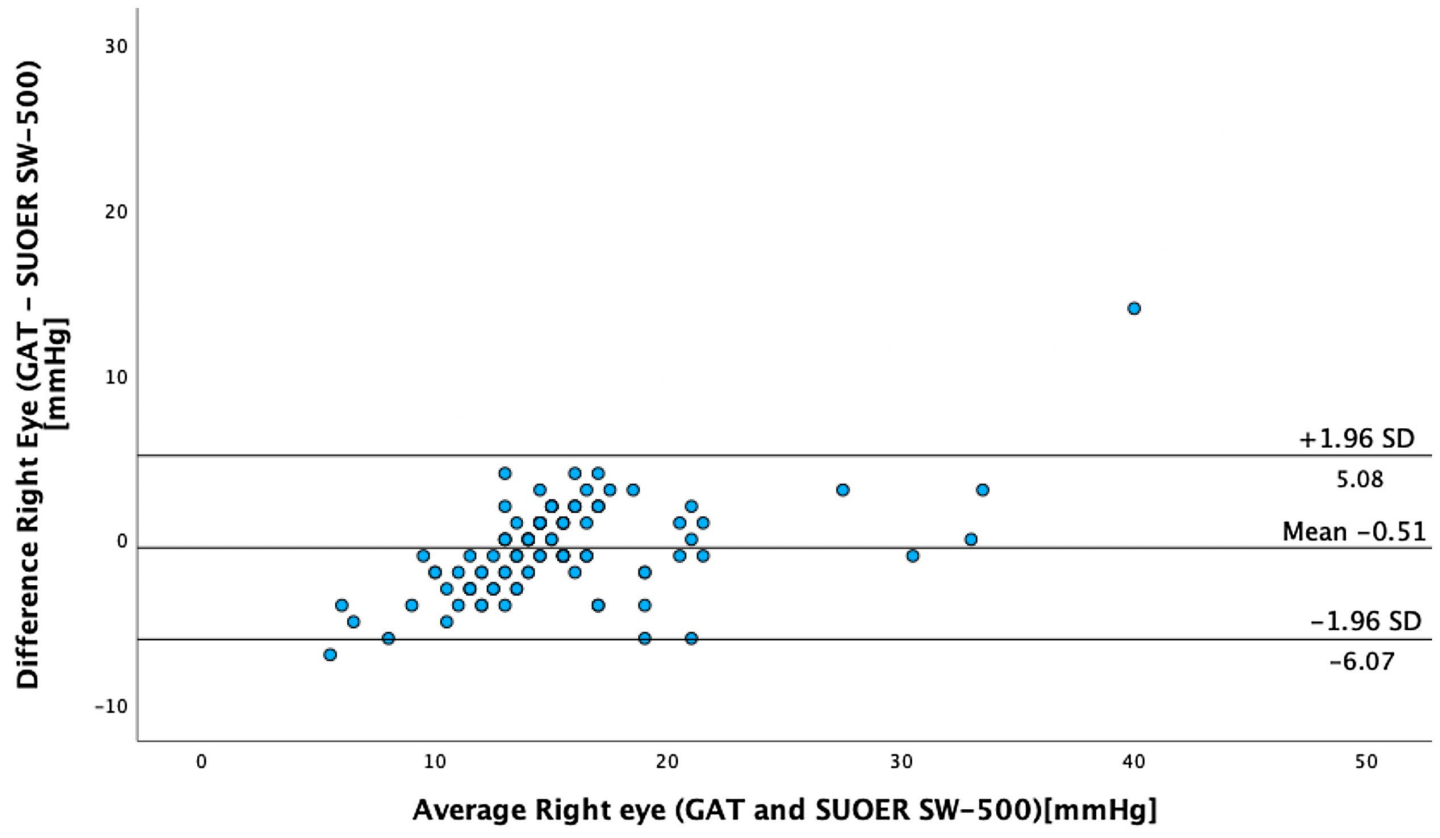
The Bland–Altman test of GAT versus SUOER SW-500 right eye.

**Figure 2:**
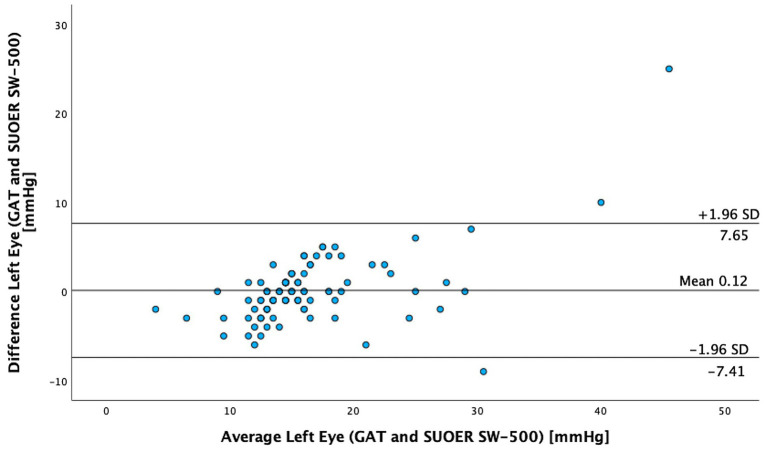
The Bland–Altman test of GAT versus SUOER SW-500 left eye.

The mean IOP of the right eye was 15.1 mm Hg (95% CI, 14.0–16.3 mm Hg) using conventional prisms and 15.6 mm Hg (95% CI, 14.8–16.5 mm Hg) with the SUOER SW-500 rebound tonometer. The mean IOP of the left eye was 16.3 mm Hg (95% CI, 14.9–17.8 mm Hg) using conventional prisms and 16.2 mm Hg (95% CI, 15.2–17.2 mm Hg) with the SUOER SW-500 rebound tonometer. The range of IOP was 2 to 58 mm Hg. Using the Bland–Altman method of differences, the SW-500 Tonometer overestimated the IOP by 0.5 mm Hg in the right eye (Figure 1) and underestimated it by 0.1 mm Hg in the left eye (Figure 2). The limits of agreement were from −6.07 to 5.08 for the right and from −7.41 to 7.65 for the left eye.

**Figure 3:**
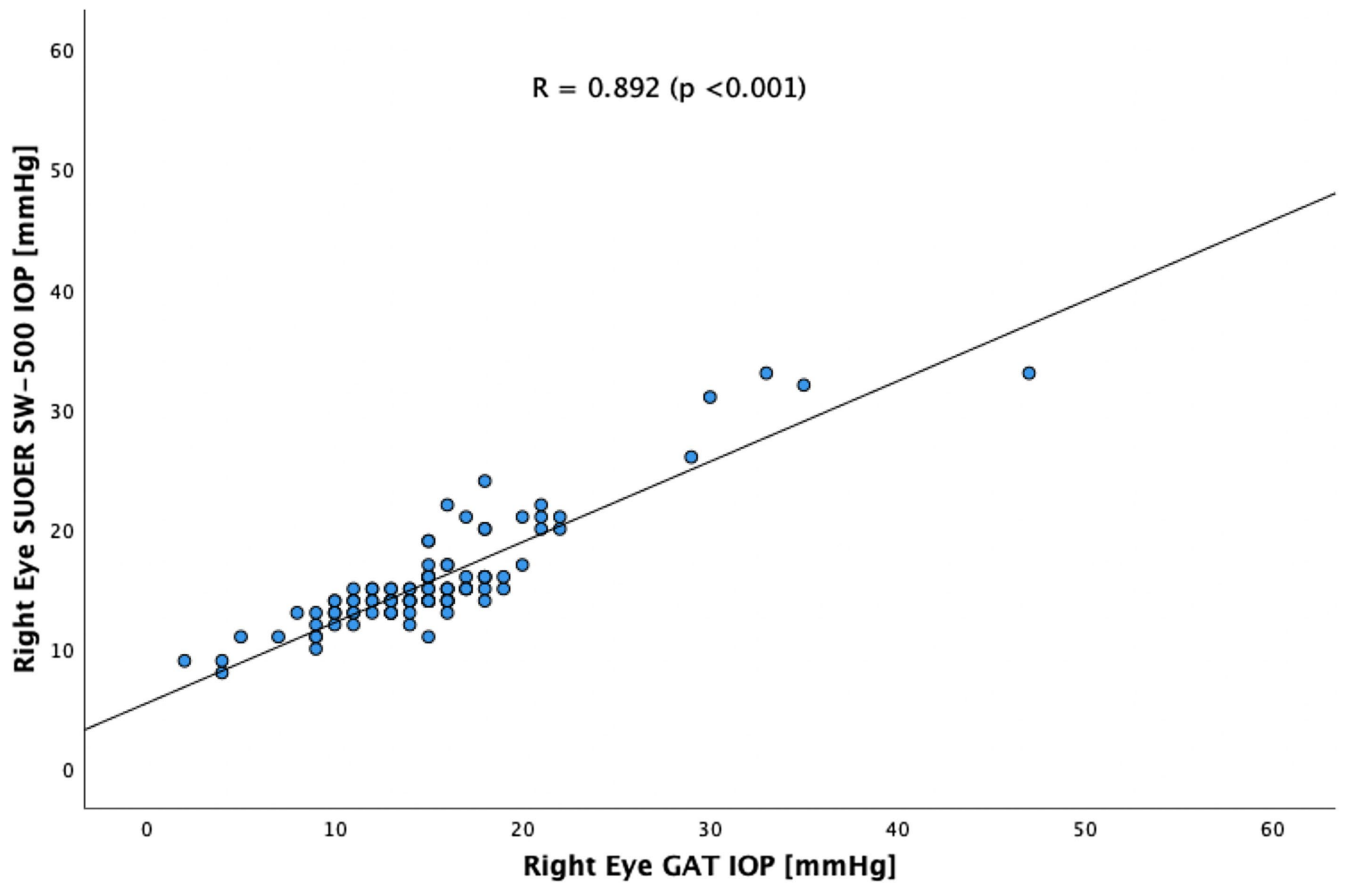
Scatter plot of the right eye SUOER SW-500 IOP against the GAT IOP.

**Figure 4:**
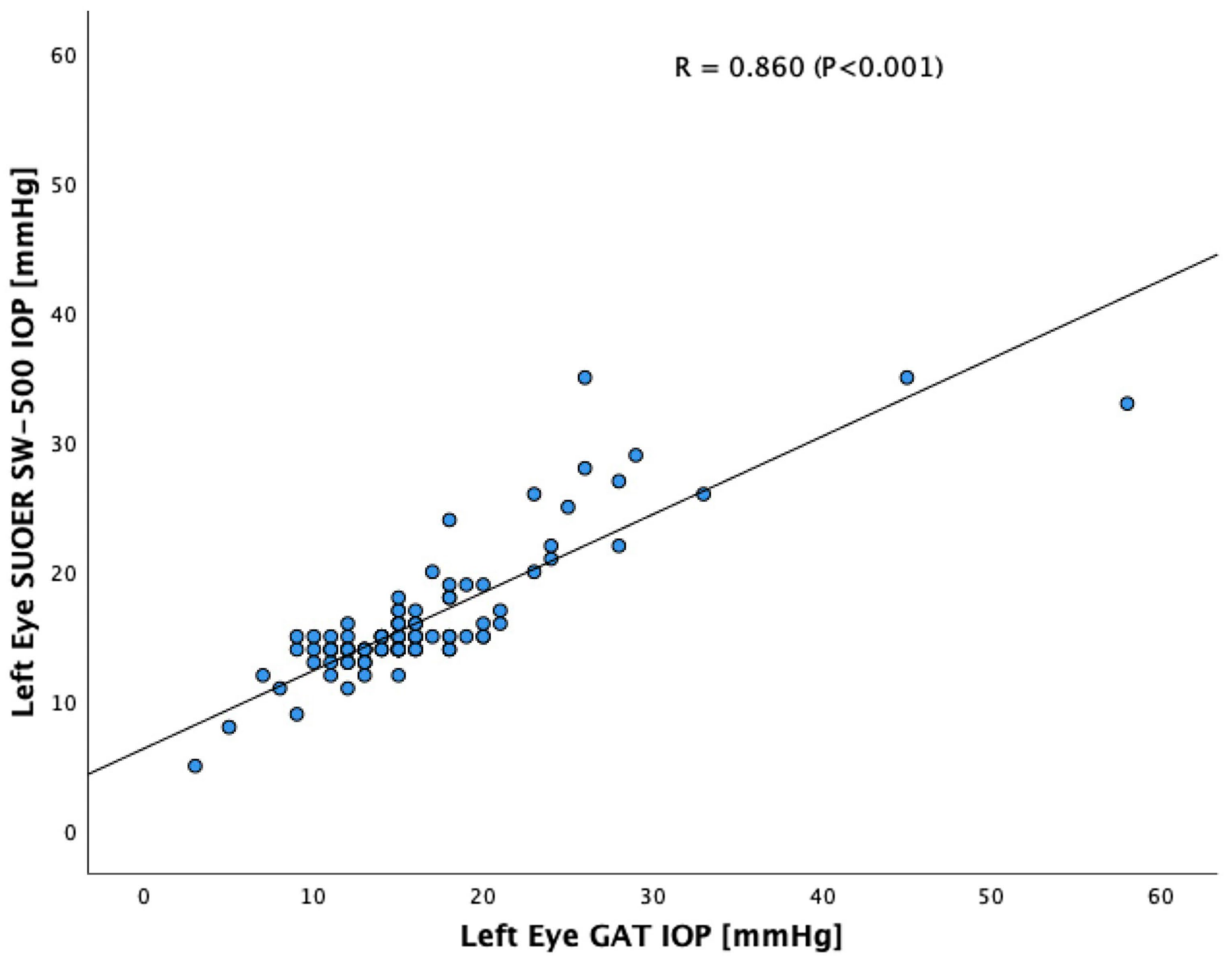
Scatter plot of the left eye SUOER SW-500 IOP against the GAT IOP.

**Figure 5:**
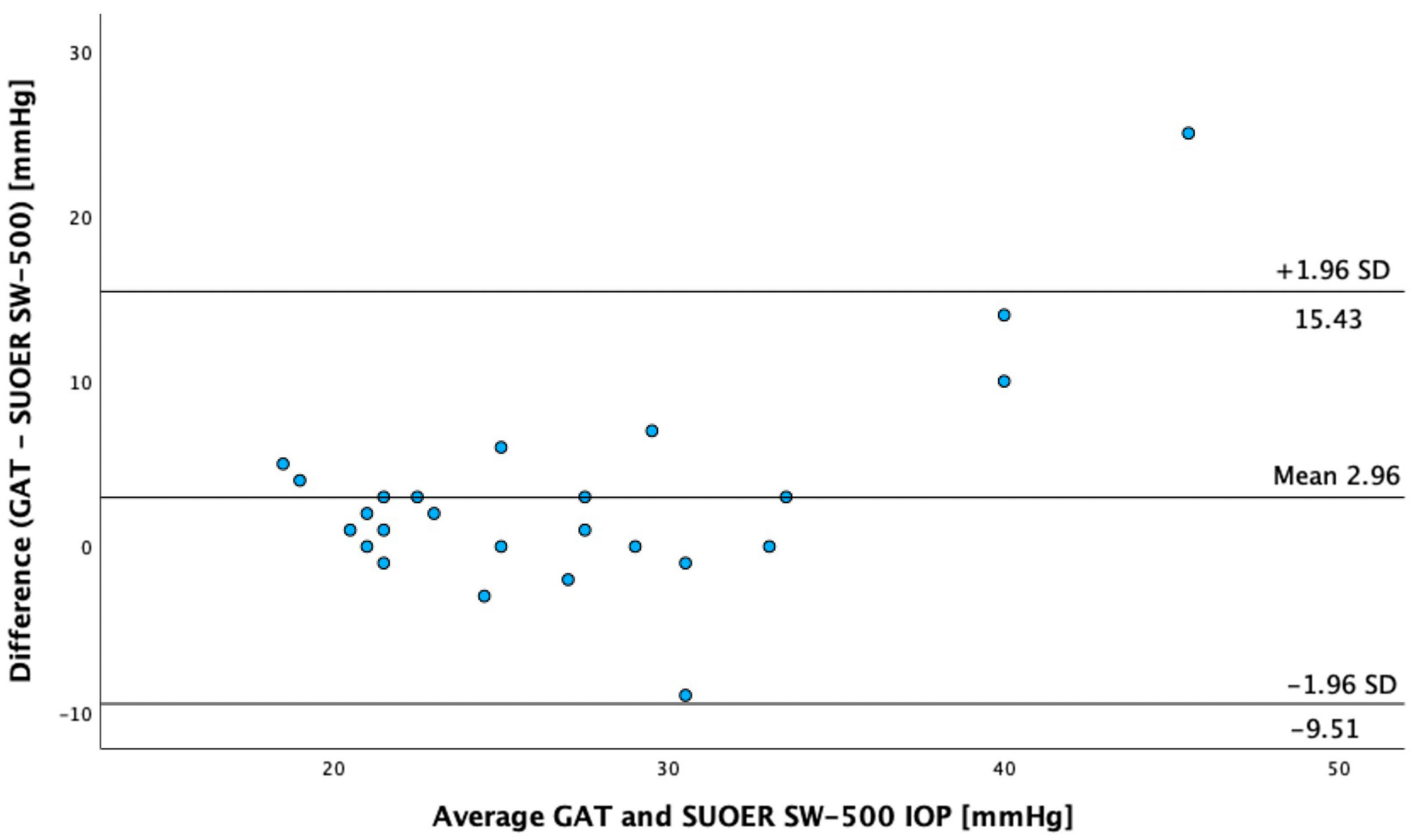
The Bland–Altman test GAT versus SUOER SW-500 (for intraocular pressure ≥21 mm Hg).

The SUOER SW-500 rebound tonometer correlated well with GAT, with a Pearson coefficient of correlation(*r*) of 0.89 (*p* < 0.001) for the right eye (Figure 3) and 0.86 (*p* < 0.001) for the left eye (Figure 4). In our cohort of 25 eyes with IOP ≥ 21 mm Hg (Figure 5), we found that the SUOER tonometer underestimated the IOP by 2.96 mm Hg with limits of agreement being from −9.51 to 15.43. Based on the SW500 product information page, the precision of this device is ±1.5 mmHg for IOPs in the range of 3 mmHg to 25 mmHg, and ± 2.5 mmHg for IOPs in the range of 25 mmHg to 70 mmHg. In our study, for patients in the IOP ≥21 group, 6 out of 10 right eyes fell within a range of ±1.5 mmHg of the GAT measurement; while 3 out of 15 left eyes, fell within the range of ±1.5 mmHg of the GAT measurement.

## Discussion

In our study of 205 eyes of 106 patients who attended glaucoma subspecialty clinics, we found that the mean difference in GAT- and SUOER SW-500 Rebound tonometry IOP measurements was between −0.1 to +0.5 mmHg. IOP correlated well with the GAT-measured IOP across a wide range of IOPs, however, the results were not interchangeable.

The SUOER RBT overmeasured IOP compared to GAT in the right and undermeasured IOP in the left eye. Of note, the right eye was measured first with GAT then RBT and then inversed for the left eye. The mean difference was +0.5 mmHg in the right eye and −0.1 mmHg in the left eye. The largest difference in IOPs occurred in eyes with IOPs at extreme ends of the spectrum. The difference in each eye could have been a result of inaccuracies of the RBT for IOPs that were very high and IOPs that were very low. For example, in one eye the GAT measured 58 mmHg but the RBT measured 33 mmHg, underestimating the IOP. At the other end of the IOP spectrum, the GAT measured 2 mmHg but the RBT measured 9 mmHg, overestimating the IOP in this case. Additionally, the right-handed operator may find it more difficult to position the device when measuring the IOP from the right eye and the patient’s nose may have been in the way resulting in less consistent measurements in the right eye compared to left eye.

There are various models of Icare in the market. The TA01i, Icare Pro, Icare ic100 and Icare ic-200 are similar in design to the SUOER RBT. However, each tonometer have different characteristics and comparative IOP value measurements. The mean difference in IOP with the SUOER RBT is smaller than those obtained in earlier studies with the Icare ic100. In a large study of 1,000 eyes reported by Subramaniam et al., the Icare consistently underestimated IOP by −4.2 mmHg (SD 4.3) (15). Other studies reported differences in mean IOP measured. In a study of 45 eyes Nakakura et al. reported a mean difference of −2.5 mmHg (SD 2.8); the same author subsequently did a larger study of 106 eyes and reported a difference of −4.2 mmHg (SD 3.0) (16, 17). This may suggest that the SUOER RBT may produce IOP measurements that are more consistent with GAT measurements as compared to the ICare ic100.

In a study of 65 eyes using the Icare TA01i by Salim et al., they reported a mean difference of +2.45 mmHg (SD 4.24). Previous studies with the Icare TA01i have also shown that it records a higher mean IOP than GAT: 3.35 ± 2.28 mmHg (18), 1.40 ± 2.19 mmHg (19), 0.6 ± 3.27 mmHg (20), and 1.49 ± 2.90 mmHg (21). The ICare Pro is the updated version of the Icare TA01i and reportedly gives a mean IOP that is closer to the GAT value. Previous studies showed that the mean IOP difference between Icare PRO and GAT was between −0.38 mmHg to 0.43 mmHg (22, 23). As for the newest model of Icare the IC200, the mean IOP was approximately 3 mmHg lower than that of GAT IOP in a recent study by Nakamura et al. (24). However, 2 other studies comparing the IC200 showed that the IC200 measured a higher IOP than the GAT (18, 25). In a study of 96 Glaucoma and 60 healthy subjects Badakere et al. reported a mean difference of +1.27 mmHg (25). Additionally, Perez-Garcia et al. (18) reported that IC200 measured 0.82 mmHg higher than GAT in a study of 40 patients with congenital glaucoma and 42 healthy subjects. There variations in IOP measurements between the Icare and GAT in previous studies described above, could have been a result of the vastly difference sample size of each study and the extremes of IOP values included. Additionally, IOP measurements by both devices could have been affected by biomechanical properties of the cornea, such as corneal thickness, curvature, underlying corneal pathologies and rigidity which were not evaluated as it was not the focus of these studies.

The SUOER SW-500 Rebound tonometry seemed more inconsistent in measuring higher IOP ≥21 mm Hg. This may limit the scope of the SUOER RBT in routine glaucoma clinics when higher IOPs are encountered. The Early Manifest Glaucoma Trial suggested that a 1 mm increase in IOP is associated with an 11% increase in the hazard ratio in the development of glaucoma (26). Similarly, previous studies on the Icare also suggest poor correlation with GAT at high IOP ranges (≥23 mmHg) (27, 28).

Although the study was conducted in glaucoma clinics, most of the IOPs measured were within the normal range. Our study cannot be applied to eyes with corneal disease or where surgery has been performed (e.g., Fuchs endothelial dystrophy, excimer laser surgery, lamellar or penetrating keratoplasty), as these patients were excluded. Additionally, the 2 groups could have been randomized to have either the right or the left eye measured first with our set protocol of IOP measurement. Other limitations of this study was the relatively small sample size of our subgroup of 25 eyes with IOP ≥ 21 mm Hg. A larger sample size of glaucoma patients with both low and high IOP values would have been ideal. We acknowledge that bilateral eyes were included but Bootstrap or generalized estimating equations were not used to account for inter-eye correlation this may have led to smaller *p*-values with eyes in the same group (29). However, we decided that our study’s primary focus is on comparing the performance of the SW500 tonometer against the GAT, rather than assessing individual patient-level variations in IOP and hence decided that the correlation between eyes of the sample patient is less relevant to our research question. Ocular parameters such as corneal thickness and axial length were not included. Additionally, our study did not test IOP with patients in the supine position which may be relevant to its future use in bedbound patients. De Bernado et al. evaluated IOP in sitting, supine, prone, and standing positions and again 5 minutes after standing, utilizing an Icare Pro (ICP) and a Tono-Pen Avia (TPA). They reported an agreement between the 2 devices which both confirmed the increase in IOP in the supine position, and also an increase after prolonged standing (30). In a study by Avitabile et al. (28) on the effects of refractive errors on IOP measurements obtained with RBT and GAT, they reported RBT readings to be >2 mm Hg in 17.9% (emmetropic), 13.3% (hyperopic), 34.5% (myopic), and 7.6% (astigmatic) eyes. Given our high prevalence of Myopia in Singapore, underlying myopia and other forms of refractive efforts may have had an effect on IOP readings by RBT.

The portability and ease of use of the SUOER Rebound tonometer makes it a potential alternative in mass eye screenings and for patient that otherwise are unable to be examined on the slit lamp. The disposable tips may help with the prevention of spread of infectious organisms. Besides adenovirus, more destructive organisms commonly implicated in contact lens-related microbial keratitis such as Pseudomonas, Staphyloccocus, and Acanthamoeba may also be spread by tonometer tips. Additionally, the SUOER SW-500 Rebound tonometer may be a good potential tool for home screening of IOP by a patient’s caregiver. Its inbuild software is able to store the last 999 IOP readings and may provide the reviewing clinician a good understanding of the IOP trend of the patient across an extended period. The Bluetooth capability of the tonometer also allows IOP measurements to be fed to the patient or caregiver’s smart phone. This may allow the patient to be more present with their management of glaucoma. In developing countries where more infectious eye diseases prevail, disposable rebound tonometer tips would confer a major advantage. The cost is also much lower than the iCare and the device is powered by 2AA batteries. In comparison to the iCare, it can also be used vertically as well as horizontally as it has an electromagnet that holds the probe in place. Additionally, it also connects to an infrared pocket printer to make hard copies of measured IOP data.

In conclusion, we have shown that the SUOER SW-500 Rebound tonometer-measured IOP correlates well with the GAT-measured IOP especially when IOP is in the normal range, but the results are not interchangeable during any transition period or from site to site. We acknowledge the SUOER SW-500 Rebound tonometer is not a substitute for GAT in the glaucoma clinic where general IOP measurements may higher. However, the SUOER SW-500 rebound tonometer presents a viable alternative to GAT in several circumstances such as where GAT measurements cannot be done, where spread of ocular infection is of concern or in the setting of mass health screenings.

## Data availability statement

The raw data supporting the conclusions of this article will be made available by the authors, without undue reservation.

## Ethics statement

The studies involving humans were approved by Singapore Eye Research Institute Institutional Review Board. The studies were conducted in accordance with the local legislation and institutional requirements. Written informed consent for participation was not required from the participants or the participants’ legal guardians/next of kin in accordance with the national legislation and institutional requirements.

## Author contributions

JC: Data curation, Formal analysis, Writing – original draft, Writing – review & editing. TS: Data Curation, Writing – review & editing. KQ: Data Curation, Writing – review & editing. SP: Investigation, Methodology, Supervision, Validation, Writing – original draft, Writing – review & editing.
